# Cognitive behavioral therapy for tobacco cessation among government bus drivers in Patna: Results from a randomized controlled trial

**DOI:** 10.6026/973206300220943

**Published:** 2026-02-28

**Authors:** Gagan Raj, Ankita Jain

**Affiliations:** 1 Department of Public Health Dentistry, Teerthanker Mahaveer Dental College & Research Centre, Teerthanker Mahaveer, University, Moradabad, Uttar Pradesh, India

**Keywords:** Cognitive behavioral therapy (CBT), basic health education, tobacco cessation, Fagerström Test for Nicotine Dependence (FTND), bus drivers, randomized controlled trial

## Abstract

The effectiveness of CBT compared to BHE for tobacco cessation remains unclear, particularly among government bus drivers in Patna. A randomized trial involving 200 smokers (aged 35-44) assessed outcomes at baseline, 4 and 12 weeks using the Fagerström Test for Nicotine Dependence (FTND), expired carbon monoxide (CO) and the Transtheoretical Model (TTM). Results showed that 60% of the CBT group quit smoking compared to 35% in the BHE group. CBT also led to a significant reduction in FTND scores and CO levels. This suggests that CBT is more effective than BHE in tobacco cessation. The study addresses the gap in understanding the comparative effectiveness of Cognitive Behavioral Therapy (CBT) and Basic Health Education (BHE) in promoting tobacco cessation among smokers.

## Background:

Tobacco use is a major contributor to preventable disease and premature death worldwide, accounting for more than 8 million deaths annually [1]. In India, smoking prevalence remains substantial, especially among men and lower- socioeconomic groups [2]. Bus drivers constitute a high-risk occupational category, frequently exposed to long working hours, stress and irregular routines [3]. According to GATS-2 (2016-17), about 25.9 % of adults in Bihar smoke [4], exceeding many state averages. Basic Health Education (BHE) programs primarily raise awareness about tobacco hazards but rarely modify psychological dependence [5]. Conversely, Cognitive Behavioral Therapy (CBT) targets maladaptive thoughts and behaviors sustaining addiction through strategies like cognitive restructuring, self-monitoring, problem-solving and relapse-prevention [6]. Prior research indicates CBT's superiority to information-only interventions [8, 9- 10]. However, its implementation among transport workers in Bihar is largely unexplored [7]. Therefore, it is of interest to evaluates and compare CBT and BHE for smoking cessation among government bus drivers in Patna.

## Materials and Methods:

A parallel, single-blind, two-arm randomized controlled trial was conducted between 2024 and 2025 at the Government Bus Stand, Patna. Ethical approval was obtained from the Institutional Ethics Committee of Teerthanker Mahaveer Dental College & Research Centre and the trial was registered at ClinicalTrials.gov (CTRI/2025/06/089460). Among 850 screened drivers, 253 met eligibility criteria and 200 consented to participate. They were randomized equally to CBT (n = 100) and BHE (n = 100). Inclusion criteria: male drivers (35-44 years), current smokers of any smoked tobacco form and willingness to attend weekly sessions. Exclusion criteria: use of smokeless tobacco, ongoing nicotine-replacement therapy, or serious systemic illness. Each group attended four weekly, group-based sessions (five participants per session; 200 minutes each) conducted by the principal investigator trained in behavioral counseling under supervision of a clinical psychologist. CBT Group: Received modules covering awareness of triggers, cognitive restructuring, coping-skills training, self-efficacy enhancement and relapse-prevention techniques.

## BHE Group:

Receive structured lectures and leaflets on harmful effects of tobacco, motivation to quit and general health messages (14). Primary Outcome: Smoking status (quit / reduced / no change) at 12 weeks verified by CO monitoring.

Secondary outcome: Change in FTND score (15).

## Behavioral measures:

The Transtheoretical Model (TTM) questionnaire (16) assessed stage of change and self-efficacy. Assessments occurred at baseline, 4 weeks and 12 weeks. CO levels (ppm) were measured using Bedfont Micro III Smokerlyzer. TTM responses were used to classify participants into pre-contemplation, contemplation, preparation, action, or maintenance stages. Analyses were performed using IBM SPSS v26. Data normality was assessed via Shapiro-Wilk test. Within-group changes were analyzed using Wilcoxon signed-rank test; between-group differences used Mann-Whitney U and Chi-square tests. Significance: p < 0.05.

## Results:

**Figure 1:**
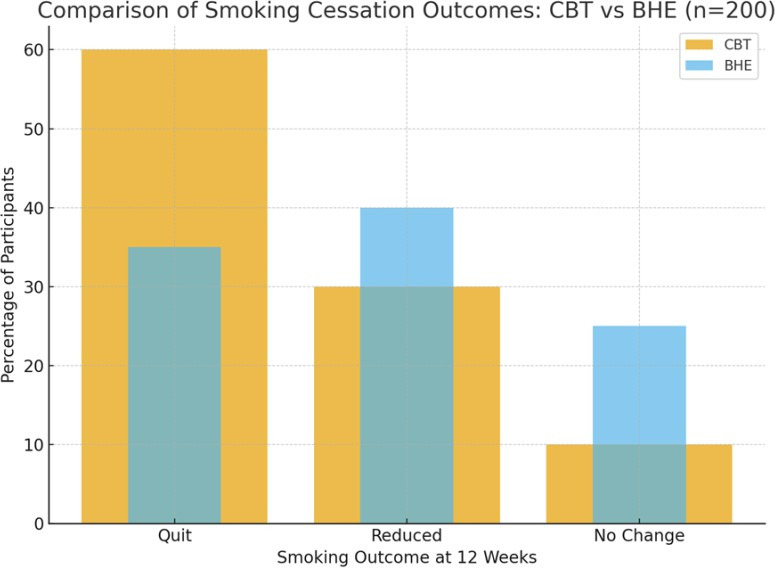
Smoking cessation outcomes (CBT versus BHE)

**Figure 2:**
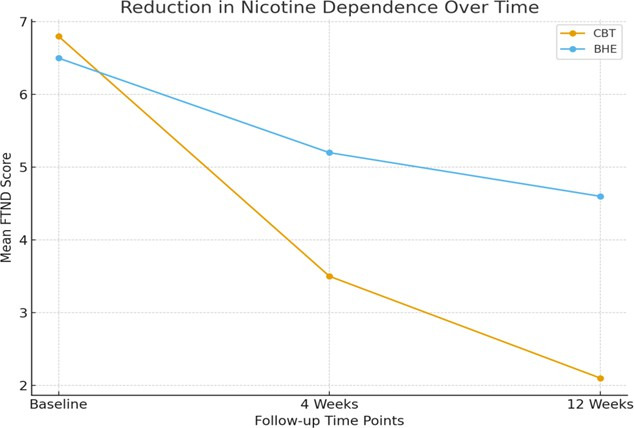
Reduction in FTND scores over time

The outcomes of smoking cessation at 12 weeks are presented in Table 1. The Cognitive Behavioral Therapy (CBT) group showed a significantly higher quit rate, with 60% (60 out of 100) of participants successfully quitting smoking, compared to the Brief Health Education (BHE) group, where only 35% (35 out of 100) achieved abstinence. A higher proportion of participants in the BHE group, 40%, reported a reduction in smoking, compared to 30% in the CBT group. In contrast, 25% of participants in the BHE group showed no change in smoking habits, while only 10% in the CBT group had no change. Figure 1 illustrates the smoking cessation outcomes for both groups, highlighting the greater success in quitting among the CBT group. Table 2 presents the change in Fagerström Test for Nicotine Dependence (FTND) scores over time. At baseline, the CBT group had a mean score of 6.8 ± 1.2, while the BHE group had a mean score of 6.5 ± 1.4. By the 4-week mark, the CBT group reduced their FTND score to 3.5 ± 0.8, showing a significant improvement. The BHE group also showed improvement, reducing their FTND score to 5.2 ± 1.1, though not as significantly. At the 12-week follow-up, the CBT group further reduced their FTND score to 2.1 ± 0.7, indicating a continued decrease in nicotine dependence, while the BHE group had a mean FTND score of 4.6 ± 1.0. Figure 2 shows the reduction in FTND scores for both groups over the 12-week period, highlighting the greater reduction in nicotine dependence for the CBT group compared to the BHE group.

## Discussion:

This trial confirms that CBT was significantly more effective than BHE in reducing nicotine dependence and facilitating cessation among bus drivers. CBT's focus on cognitive restructuring and coping strategies addresses the psychological roots of tobacco use [8]. Similar trials in India and abroad report improved quit rates with. The enhanced FTND improvement and CO reduction reflect increased self-efficacy and motivation observed through TTM shifts [9]. In contrast, BHE's didactic approach relies on information transfer without behavioral reinforcement [10]. Behavioral skills such as identifying triggers and coping with cravings are better internalized in CBT sessions. Results mirror findings among factory workers and transport staff in Taiwan and Thailand and further research by Raj *et al.* (2025) [11] highlights the importance of behavioral interventions in health-related outcomes.

## Limitations:

[1] Single-center urban study (Patna); results may not generalize to other occupational groups.

[2] Short follow-up (12 weeks) without biochemical cotinine confirmation.

[3] Possible reporting bias despite objective CO measurement.

## Implications:

Integration of CBT modules into state transport wellness and National Tobacco Control Program (NTCP) frameworks could enhance long-term cessation success. Future studies should evaluate cost-effectiveness and sustainability over ≥ 6 months (25).

## Conclusion:

Cognitive Behavioral Therapy proved significantly more effective than Basic Health Education in achieving abstinence and reducing nicotine dependence among government bus drivers in Patna. CBT's structured, behavior-focused framework makes it a promising component for occupational health initiatives targeting tobacco control in India.

## Figures and Tables

**Table 1:** Smoking cessation outcomes at 12 weeks

OUTCOME	CBT(N=100)	BHE(N=100)
QUIT	60(60%)	35(35%)
REDUCED	30(30%)	40(40%)
NO CHANGE	10(10%)	25(25%)

**Table 2:** Change in FTND scores

Group	Baseline Mean	4 Weeks	12 Weeks
CBT	6.8 ± 1.2	3.5 ± 0.8	2.1 ± 0.7
BHE	6.5 ± 1.4	5.2 ± 1.1	4.6 ± 1.0
